# Vulcanizers and Vulcanizing

**Published:** 1882-06

**Authors:** A. Berry


### VULCANIZERS AND VULCANIZING.

BY A. BERRY, D.D.S.

Your patient unceremoniously tells you she wants a set of teeth on rubber. You scrutinize her. You think it possible to persuade her to have something better than rubber

You expatiate on the strength and comfort of gold as a base for teeth. You paint in glowing colors, as you justly may, the intrinsic beauty of continuous gum. You explain to her the advantages of silver for temporary purposes, and for impecunious persons. You depict in strong language the clumsiness, want of strength and ill looks of rubber.

If you see that you failed to gain your point, you call up the forlorn hope, and describe, with alarming eloquence, the imminent danger of poisoning from sulphuret of mercury in the rubber. You relate pathetically the particulars of some distressing cases that have come under your observation. You rest now from your unselfish efforts. You look confidently for your patient to assent to the importance of your utterances.

You look in vain. With perfect nonchalance she tells you that a dozen persons of her acquaintance wear teeth on rubber, and have no trouble with it. She reasserts her preference for rubber. You feel intensely disgusted. You hush up. You wish the rubber was all in Para. But you submit. There is nothing else for you to do.

Do what you may, the indomitable rubber confronts you on every hand. It has invaded all ranks of society. The high, the low, the rich, the poor, the learned and the illiterate, are alike its votaries. It has come to stay.

In this connection a few remarks on vulcanizers, and their working, may not be out of place.

The improvements in dental vulcanizers are quite gratifying, from the original with its numerous bolts and screws, advertised to weigh only forty pounds, to those ot the present day five pounds weight, and some of them secured by only one screw, requiring little time to fasten safely.

For packing, asbestus is better than rubber, as it is less liable to allow the escape of steam, and lasts much longer. When used the first time it may be dusted over with pulverized steatite, to prevent it adhering to the top.

It is advantageous to have a pin in the rim of the common vulcanizer, adapted to a hole or notch in the top, to insure a fit without variation at different times of closing as well as to save the annoyance of looking at marks.

A thermometer attached to a vulcanizer is a superfluity. The degree of heat may be known more readily, and with sufficient accuracy, after a little experience, by placing a drop of water on some prominent part, watching the boiling point.

A vulcanizer should never be used without a safety valve. The most careful persons are sometimes liable to forget to watch properly. Nearly all the explosions of vulcanizers might have been prevented by safety valves.

It is well to raise the heat of the vulcanizer slowly and steadily in about two hours to 320 degrees, and hold it there about twenty minutes, and then to drop the heat and allow the instrument to become cold before opening. A little practice will afford the requisite knowledge as to time to keep the heat at 320 degrees. In this manner rubber is much stronger than when hardened in the usual shorter time.

